# A quest to unravel idiopathic ventricular fibrillation

**DOI:** 10.1007/s12471-024-01874-8

**Published:** 2024-05-07

**Authors:** Joris R. de Groot

**Affiliations:** 1 Department of Cardiology, Heart Centre, Amsterdam University Medical Centres/University of Amsterdam, Amsterdam, The Netherlands; 2 Amsterdam Cardiovascular Sciences, Heart Failure and Arrhythmias, Amsterdam, The Netherlands

Ventricular fibrillation (VF) is the most common mechanism of sudden cardiac death (SCD). The context in which VF occurs may vary, but coronary artery disease, in particular acute myocardial infarction, and cardiomyopathies are responsible for the vast majority of VF cases. Consequently, a survivor of VF undergoes several diagnostic procedures to uncover the cause of the cardiac arrest and is treated accordingly. Many patients who survive SCD may need an implantable cardioverter-defibrillator (ICD) for secondary prevention, and vice versa, the wider uptake of ICDs for primary or secondary prevention has reduced the proportion of VF cases among out-of-hospital cardiac arrest victims [1]. Yet, despite comprehensive diagnostic testing, usually comprising coronary angiography, echocardiography, cardiac magnetic resonance imaging, a small proportion of VF cases, estimated at < 5%, remain unresolved. These cases are referred to as idiopathic VF. As idiopathic VF is a diagnosis by exclusion, the definition can only be used in cases where comprehensive investigation has indeed taken place, albeit this is not always done in different studies.

Moreover, idiopathic VF is not a static condition, and improving imaging techniques, the discovery of new genetic entities, and intoxication with novel designer party drugs may turn idiopathic VF cases into a diagnosis. Nevertheless, and despite this progress, it remains extremely important to investigate the causes and to identify novel mechanisms of disease, to advance the treatment of VF patients.

In this issue of the *Netherlands Heart Journal*, Verheul and colleagues present a progress report on the Dutch Idiopathic Ventricular Fibrillation Registry [2]. The outline of the registry has been published previously in this journal [3]. Eligible patients (*n* = 432 from 11 hospitals across the Netherlands) had VF or polymorphic ventricular tachycardia as the presenting rhythm. Established cardiac, respiratory, metabolic or toxicological aetiologies of VF were exclusion criteria. The minimum set of diagnostic tests for evaluation of the cause of VF consisted of an initial evaluation through assessing history, family history, physical examination, blood chemistry and toxicological screening. Next, electrocardiography, echocardiography and coronary angiography were part of the initial screening procedure. When these tests did not yield a diagnosis, the cardiac arrest was deemed unexplained, and further electrocardiographic tests including Holter monitoring and exercise electrocardiogram (ECG) testing were performed, as well as advanced imaging with cardiac magnetic resonance. Finally, sodium channel blocker provocation, ergonovine provocation testing for coronary spasm and targeted genetic testing may be performed. Verheul et al. outline that these test strategies were performed before inclusion in the registry in 63–100% of cases, with the exception of toxicological screening (15%) and ergonovine provocation (19%).

During follow-up of the patients in the registry, a definitive diagnosis was established in a mere 38 patients (9%) during 11 years of follow-up, lower than the 22% that was reported in the initial phase of the registry [3]. Most diagnoses established during follow-up were cardiomyopathies (arrhythmogenic cardiomyopathy in 9 patients (24%), dilated cardiomyopathy in 5 (13%) and hypertrophic cardiomyopathy in 4 (10%)), channelopathies (Brugada syndrome in 5 patients (13%), long QT syndrome in 1 (3%), catecholaminergic polymorphic VT in 5 (13%) and early repolarisation syndrome in 3 (8%)). Further diagnoses were myocarditis (*n* = 1, 3%), coronary artery spasm (*n* = 2, 5%) and others (*n* = 3, 8%).

Three thoughts come to mind. The first is that several of the definitive diagnoses outlined above could potentially have been made before the VF case was deemed idiopathic. The second underscores the relevance of diligent follow-up, and detection of the unfolding of the arrhythmogenic (or structural) substrate of VF. Evidently, patients with idiopathic VF need to be protected from recurrent events with an ICD, but detection of the cause of their index event will allow tailored treatment, and potentially risk stratification in family members. Advanced diagnostics, including body-surface mapping ECGs, isoproterenol and Valsalva testing, may identify more causes of idiopathic VF [4]. Indeed, in order to be able to apply such strategies a registry such as that of Verheul et al. is needed for identification of VF cases, as well as close clinical follow-up and the application of novel electrocardiographic techniques and imaging. It is of interest to see that 61% of subjects in the registry are male. Whether this constitutes a selection bias of patients included in the registry, or whether true idiopathic VF is less common in females than in males, is not mentioned by Verheul et al. and may be a topic for future investigation.

Finally, the vast majority of VF cases are still awaiting a diagnosis (394 patients with a median follow-up of 6 years). This not only results in uncertainty for the patients concerned, but the 25% appropriate ICD discharge is also alarming. Apparently, when the mechanism of their ventricular arrhythmias is unknown, these patients are at high risk of recurrent events. Hence, a registry such as the idiopathic VF registry is very relevant despite its hypothesis-free and observational nature. Only through establishing a diagnosis first can therapeutic actions to minimise future risk be employed.

## References

[1] Hulleman M, Berdowski J, de Groot JR (2012). Implantable cardioverter-defibrillators have reduced the incidence of resuscitation for out-of-hospital cardiac arrest caused by lethal arrhythmias. Circulation.

[2] Verheul LM, Groeneveld SA, Stoks J (2024). The Dutch Idiopathic Ventricular Fibrillation Registry: progress report on the quest to identify the unidentifiable. Neth Heart J.

[3] Blom LJ, Volders PGA, Wilde AA, Hassink RJ (2018). Life-long tailoring of diagnosis and management of patients with idiopathic ventricular fibrillation-future perspectives in research. Neth Heart J.

[4] Haïssaguerre M, Duchateau J, Dubois R (2020). Idiopathic ventricular fibrillation: role of Purkinje system and microstructural myocardial abnormalities. JACC Clin Electrophysiol.

